# Epigenetic weapons of plants against fungal pathogens

**DOI:** 10.1186/s12870-024-04829-8

**Published:** 2024-03-06

**Authors:** Justyna Mierziak, Wioleta Wojtasik

**Affiliations:** Department of Genetic Biochemistry, Faculty of Biotechnology, University of Wroclaw, Przybyszewskiego 63, Wroclaw, 51-148 Poland

**Keywords:** Plant defense, Fungal infection, Epigenetic mechanisms, Immunity, Epigenetic memory

## Abstract

In the natural environment, plants face constant exposure to biotic stress caused by fungal attacks. The plant’s response to various biotic stresses relies heavily on its ability to rapidly adjust the transcriptome. External signals are transmitted to the nucleus, leading to activation of transcription factors that subsequently enhance the expression of specific defense-related genes. Epigenetic mechanisms, including histone modifications and DNA methylation, which are closely linked to chromatin states, regulate gene expression associated with defense against biotic stress. Additionally, chromatin remodelers and non-coding RNA play a significant role in plant defense against stressors. These molecular modifications enable plants to exhibit enhanced resistance and productivity under diverse environmental conditions. Epigenetic mechanisms also contribute to stress-induced environmental epigenetic memory and priming in plants, enabling them to recall past molecular experiences and utilize this stored information for adaptation to new conditions. In the arms race between fungi and plants, a significant aspect is the cross-kingdom RNAi mechanism, whereby sRNAs can traverse organismal boundaries. Fungi utilize sRNA as an effector molecule to silence plant resistance genes, while plants transport sRNA, primarily through extracellular vesicles, to pathogens in order to suppress virulence-related genes. In this review, we summarize contemporary knowledge on epigenetic mechanisms of plant defense against attack by pathogenic fungi. The role of epigenetic mechanisms during plant-fungus symbiotic interactions is also considered.

## Background

Due to their sedentary lifestyle, plants have to cope with constantly changing environmental conditions and also have to defend themselves against many biotic aggressors that threaten their development and reproduction. The response to various biotic stresses largely depends on the ability of the plant to rapidly modulate the transcriptome. External signals are translocated to the nucleus to activate transcription factors, resulting in increased expression of specific sets of defense-related genes. Among the mechanisms of transcription regulation in the plant response to stress, chromatin rearrangement (remodeling) emerges as a key process, based on modifications of histones by appropriate enzymes (deacetylases, acetylases, demethylases, methylases, enzymes carrying out ubiquitination) and ATP-dependent chromatin remodeling complexes. In addition, DNA methylation and non-coding RNAs also play an important role in modulating defense against stress factors.

### Plant defense against fungal pathogens

The first line of plant defense against attack by pathogens is structural resistance, conditioned by the anatomical structure of the plant. The cell wall, made of polysaccharide (cellulose, hemicellulose and pectin) and non-polysaccharide (lignin) polymers and structural proteins and enzymes [1], constitutes the external barrier of plants against pathogen infections. The second line of defense is based on the production of constitutive secondary metabolites that are harmful to pathogens and activation of the hypersensitive response (HR), which is a mechanism employed by plants to prevent the spread of infection. The HR is characterized by the rapid death of cells in the local area surrounding the infection. The third line of defense is systemic acquired resistance (SAR) and induced systemic resistance (ISR). Plants often acquire systemic resistance to further infection after local infection with a pathogen [2, 3]. This requires accumulation of the plant hormone salicylic acid in tissue distal to the site of infection, which is part of the SAR, while the ISR is associated with JA/ET-dependent signaling [2]. ISR does not induce pathogenesis-related protein synthesis but can be induced by PAMP. Mobile immune molecules can be transferred from infected areas where a defense reaction has already started to distant healthy areas, triggering SAR there to protect the whole plant from infection [4]. These molecules affect the induction of pathogenesis-related genes (PR genes) and reprogram the cell to defend itself. Proper transcription reprogramming is believed to be critical for priming and plant defense against multiple pathogens [5–7].

The main goal of pathogens when infecting plants is to obtain nutrients necessary for their growth. For this purpose, in the first phase of infection, pathogens secrete plant cell wall-degrading enzymes (CWDE): polygalacturonases (PG), pectin lyases (PL) and pectin methylesterases (PME), as well as cellulases and hemicellulases [2]. In turn, the degradation of the plant cell wall, on the one hand, enables penetration and colonization of the host by pathogens, and on the other hand, as a result of the action of polygalacturonases secreted by pathogens, oligogalacturonans (pectin fragments) are released, which act as elicitors and activate plant defense mechanisms [8–11].

Pathogen-triggered molecular defense strategies of plants that lead to activation of the immune response and the signaling cascade have been divided into two main groups. The first of them is based on the perception of pathogen- and/or microbial-associated molecular patterns or damage-associated molecular patterns (PAMPs, MAMPs, DAMPs) and uses the recognition of a pathogen through plant cell surface anchored pattern recognition receptors (PRRs). Such recognition triggers a range of downstream defense mechanisms that lead to activation of pattern-triggered immunity (PTI). Examples of fungal MAMPs that interact with PRRs are chitin, chitosan, β-glucans, elicitins and ergosterol [12–16].

The second branch utilizes the recognition of microbial effectors or their activity, through resistance proteins (R proteins) to initiate effector-triggered immunity (ETI). Plant R genes typically encode intracellular receptors with nucleotide-binding leucine-rich repeat (NB-LRR) domains, organized with central NB and C-terminal LRRs. These NB-LRR-containing R proteins (NLRs) can be further classified into Toll/Interleukin1 receptor-like (TIR) or coiled-coil (CC) types according to their N-terminal sequences. NLRs can interact directly or indirectly with pathogen effectors to induce defense responses [12, 17–20]. It is generally accepted that, in contrast to PTI, ETI induces stronger and longer-lasting responses, and leads to a hypersensitive response (HR) resulting in synthesis of pathogenesis-related proteins (PR proteins). If this response fails, programmed cell death is induced [21, 22]. The other R gene-mediated resistance response is an oxidative burst that rapidly produces reactive oxygen species (ROS), which may have a direct antifungal effect or be a signal to activate other defense responses.

**Fig. 1:**
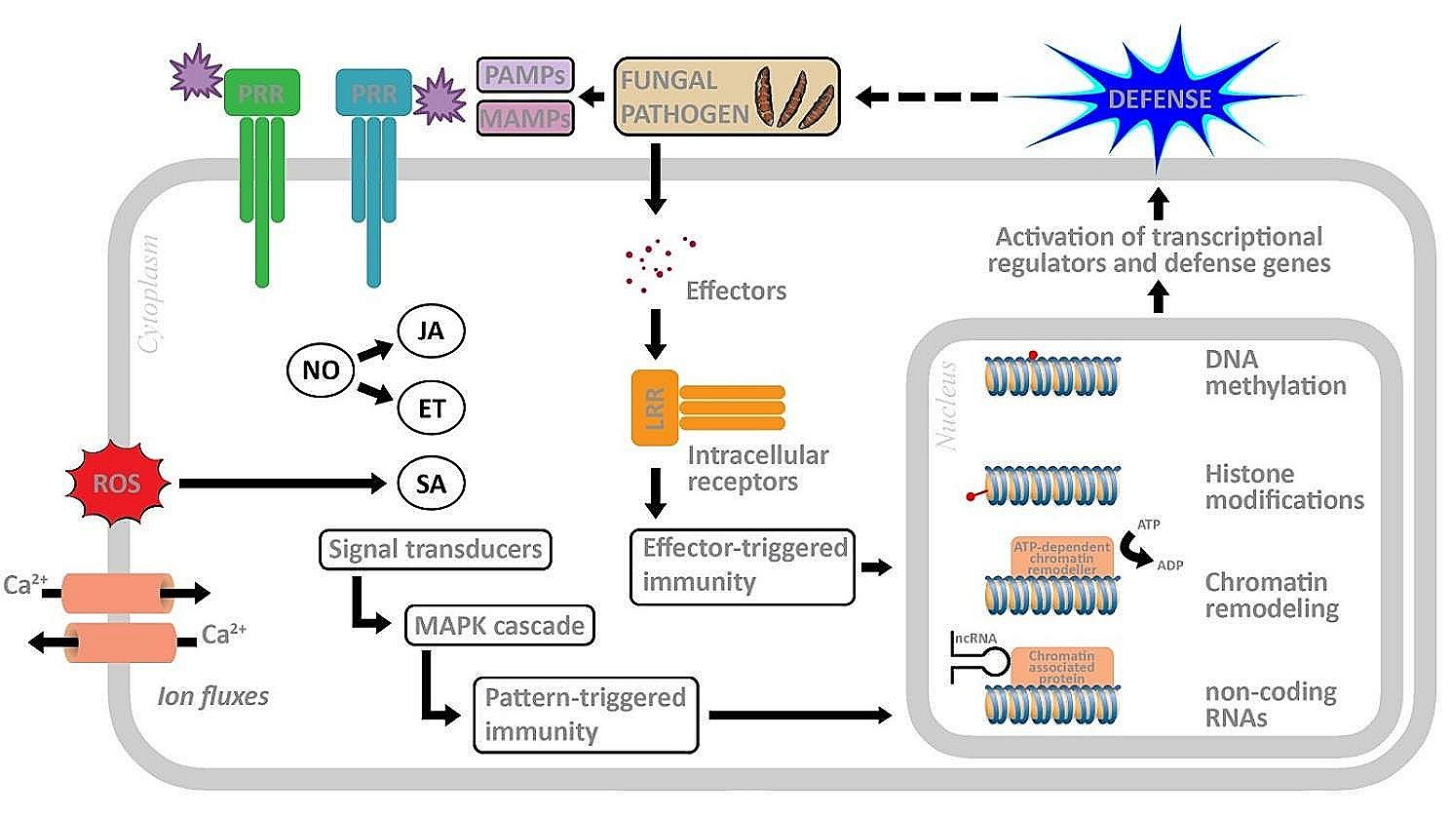
Induction of epigenetic changes involved in plant defense. Microbe-associated molecular patterns (MAMPs) and pathogen-associated molecular patterns (PAMPs) elicit pattern-triggered immunity (PTI), whereas effectors initiate effector-triggered immunity (ETI), thereby initiating intricate signaling cascades that subsequently lead to epigenetic modifications such as DNA methylation, histone modifications, chromatin remodeling, and involvement of non-coding RNAs. These modifications activate transcriptional regulators and defense genes, consequently instigating defense responses. JA, jasmonic acid; ET, ethylene; SA, salicylic acid; NO, nitric oxide; ROS, reactive oxygen species; LRR, receptors with a leucine-rich nucleotide binding site; MAPK, mitogen-activated protein kinase

PTI and ETI affect the inhibition of pathogen multiplication by inducing various immune responses, including calcium ion signaling, nitric oxide and ROS production, alteration in membrane trafficking, activation of defense genes, cell death program limitation of nutrient transfer from the cytoplasmic matrix to the apoplast, biosynthesis of antimicrobial metabolites and defense phytohormones (salicylic acid (SA), jasmonic acid (JA) and ethylene (ET)), hydrolytic enzyme production, callose deposition at the infection site, stomatal closure and activation of the mitogen-activated protein kinase (MAPK) cascade, which leads to activation of WRKY type transcription factors, which activated by phosphorylation bind to the W-cassette of promoters of pathogenesis-related genes (PR genes), and changes in defense gene expression [23–26]. Epigenetic mechanisms such as DNA methylation, histone modifications and chromatin remodeling as well as epigenetic elements such as non-coding RNA contribute to modulation of gene transcription (Fig. 1) [27].

### Histone modification

**Fig. 2:**
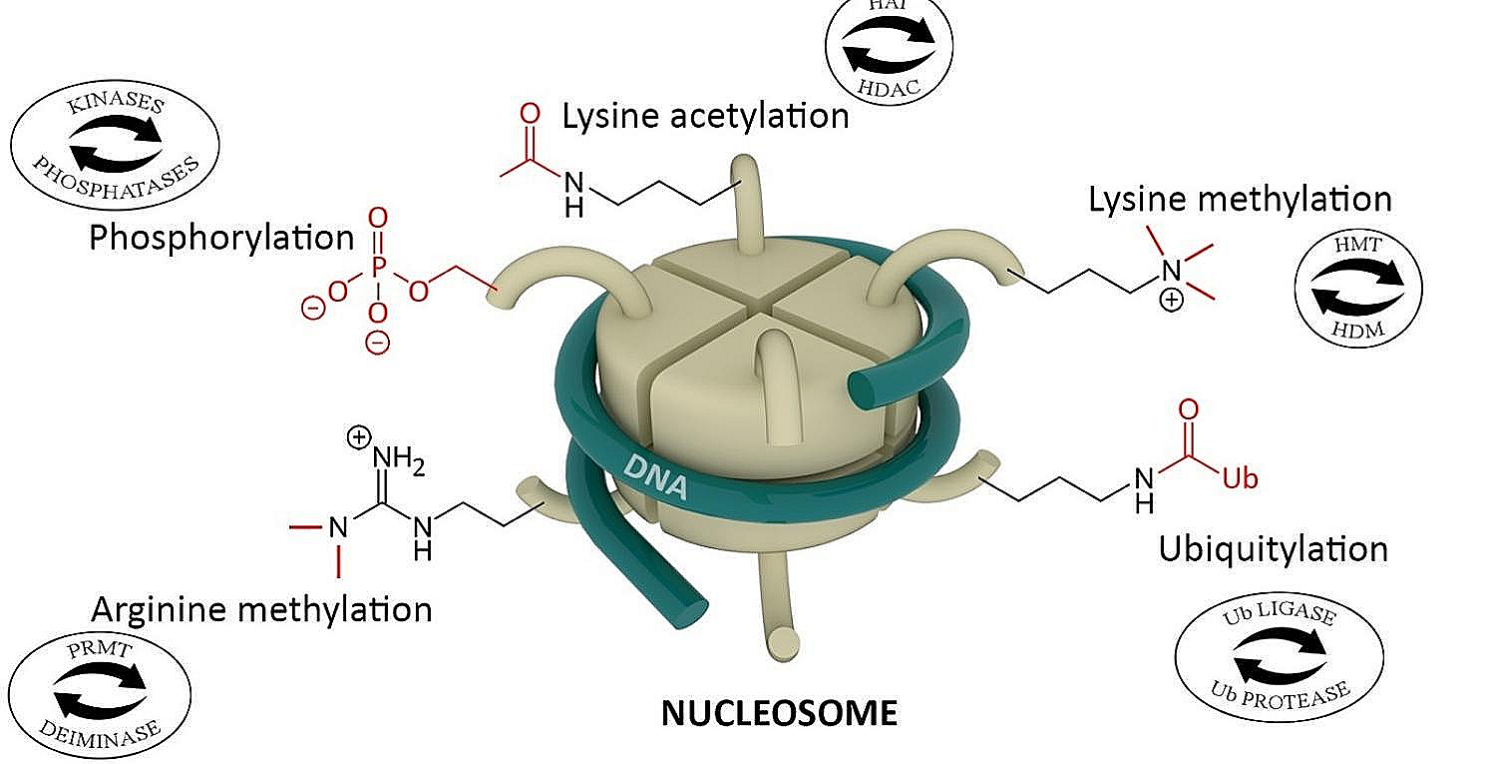
Covalent modifications of histones in plants. They include acetylation reactions catalyzed by histone acetylase (HAT); deacetylation by histone deacetylase (HDAC); methylation by histone methyltransferase (HMT) or protein arginine methyltransferase (PRMT); demethylation by histone demethylase (HDM) or deiminase; phosphorylation by kinase; dephosphorylation by phosphatase; ubiquitination by UB ligase; deubiquitination by UB protease

Gene expression is regulated by changes in chromatin structure. Less condensed regions of chromatin are transcriptionally active. Histones constituting the core of chromatin are subject to many modifications, e.g. acetylation, methylation, or ubiquitination (Fig. 2).

Histone acetylation disrupts interactions between nucleosomes, which leads to a looser state of chromatin and allows proteins involved in transcription to bind. In plants, lysine acetylation at the amino terminus of histone H3 and H4 tails has been linked to gene activation [28]. This modification reduces the ionic interaction between the positively charged lysine side chains and the negatively charged DNA backbone. Furthermore, lysine acetylation provides docking sites for transcriptional coactivator proteins containing bromodomains [29]. Histone acetyltransferases (HATs) are responsible for attaching the acetyl group of acetyl-CoA to the lysine amino group of histones, while histone deacetylases (HDACs) remove the acetyl group [27].

Both histone acetylation and deacetylation are involved in the plant response to pathogenic infections. Pathogen effectors can inhibit the activation of plant defense genes by e.g. interfering with the HAT function. During research on mechanisms underlying tomato responses to *Botrytis cinerea*, there was observed an increase in the H3K9 acetylation mark along the early induced genes *SlyDES, SlyDOX1, and SlyLoxD* encoding oxylipin-pathway enzymes, and *SlyWRKY75* coding for a transcriptional regulator of hormonal signaling [30]. Song et al. identified the wheat histone acetyltransferase TaHAG1 as a positive regulator of resistance to powdery mildew caused by *Blumeria graminis* f. sp. *tritici* (Bgt). TaHAG1 acts as an epigenetic modulator and physically interacts with TaPLATZ5, a plant-specific zinc-binding protein. TaPLATZ5 directly binds to the promoter of TaPAD4 and together with TaHAG1 to potentiate the expression of TaPAD4 by increasing the levels of histone H3 acetylation. Acetylation can regulate expression of the key transducer gene TaPAD4 and promote accumulation of SA and reactive oxygen species for resistance to Bgt infection. A core transducer including the PAD4-EDS1 node is proposed as a convergence point between PTI and ETI [31].

One deacetylase that plays a role in immunity is histone deacetylase 19 (HDA19). HDA19 responds to injury and JA action. *HDA19* overexpression in *Arabidopsis thaliana* (35S:HDA19) plants was associated with decreased histone acetylation levels, while *HDA19* repressed (HDA19-RNAi) plants had increased histone acetylation levels. Compared to wild-type plants, the 35 S:HDA19 transgenic plants were more resistant to the pathogen *Alternaria brassicicola*. In these plants, increased expression of genes related to pathogenesis (glucanases and chitinases) was noted, supporting a role for HDA19 in the ethylene and JA-mediated defense response. The expression of these genes was downregulated in HDA19-RNAi plants [32]. HDA19 can bind directly to PR promoters and then deacetylate histones, ensuring low expression of PR genes under normal conditions and also avoiding overexpression of these genes during a defense response [33]. HDA6 can also inhibit the expression of pathogen-responsive genes, is induced by treatment with JA and an ET precursor, and interacts with the F-box protein, coronatine insensitive 1 (COI1), which mediates JA signaling [32, 34].

The histone deacetylase TaHDT701 was identified as a negative regulator of wheat defense responses. TaHDT701 associates with the RPD3 type histone deacetylase TaHDA6 and the WD40-repeat protein TaHOS15 to constitute a histone deacetylase complex. Knockdown of *TaHDT701*, *TaHDA6*, and *TaHOS15* resulted in enhanced wheat powdery mildew resistance, suggesting that this histone deacetylase complex negatively regulates wheat defense responses. This was accompanied by increased histone acetylation and methylation [35]. Similar studies were performed on rice, where the transcription of *H4-HDT701* of rice, a member of the HD2 deacetylase family, was increased in the compatible reaction and decreased in the incompatible reaction after infection by the fungal pathogen *Magnaporthe oryzae*. Overexpression of this deacetylase in transgenic rice decreased H4 acetylation levels and increased plant susceptibility to *M. oryzae*. HDT701 silencing led to increased levels of H4 acetylation and overexpression of the pattern recognition receptor (PRR) and other defense genes. Silenced transgenic lines showed increased *M. oryzae* as well as *Xanthomonas oryzae* pv *oryzae* (Xoo) resistance. HDT701 can also bind to defense-related genes to regulate their expression. The authors conclude that HDT701 negatively regulates innate immunity by modulating the levels of histone H4 acetylation of PRR and defense-related genes in rice [36].

Research on this deacetylase in wheat was carried out by the team of Zhi et al., who identified it as a negative regulator of wheat defense responses to *Blumeria graminis* f. sp. *tritici* (Bgt). They observed that TaHDT701 associated with the RPD3 type histone deacetylase TaHDA6 and the WD40-repeat protein TaHOS15 to constitute a histone deacetylase complex. Knockdown of this complex resulted in enhanced wheat powdery mildew resistance. In the absence of the TaHDT701-TaHDA6-TaHOS15 histone deacetylase complex, chromatin at the defense-related genes resides in a primed gene expression state marked by increased H4K16Ac, H3K9Ac, and H3K4me3, as well as reduced nucleosome occupancy, thereby stimulating defense-related transcription and defense responses to Bgt [35].

The HDAC proteins in the Sir2 family, which are NAD+-dependent HDACs, play various roles in plant physiological processes. In rice, *OsSRT1* RNA interference induced an increase of histone H3K9 (lysine-9 of H3) acetylation and a decrease of H3K9 dimethylation, leading to H_2_O_2_ production, DNA fragmentation, cell death, and lesions mimicking plant hypersensitive responses during interactions with pathogens. Chromatin immunoprecipitation assays showed that *OsSRT1* down-regulation induced histone H3K9 acetylation on the transposable elements and some of the hypersensitive response-related genes [37].

Research by Mengel et al. on *A. thaliana* indicated that NO also affects histone acetylation by targeting and inhibiting histone deacetylase complexes, resulting in hyperacetylation of specific genes, e.g. involved in plant defense against pathogens. Furthermore, salicylic acid, which is the major plant defense hormone against biotrophic pathogens, inhibited HDAC activity and increased histone acetylation by inducing endogenous NO production [38].

An important chromatin modifier that plays a role in histone acetylation and plant immunity is the Elongator complex. The Elongator complex is a large multi-subunit complex involved in one of the transcription stages – elongation – and many physiological processes in eukaryotes. The results of Defraia et al. indicate that the entire Elongator complex, in *A. thaliana*, is involved in basal immunity and ETI, but not in SAR, and suggest that it may play a role in facilitating transcriptional induction of defense genes through alterations to their chromatin, such as histone acetylation. The authors described Elongator Subunit 2 (AtELP2) as an accelerator of immune responses in *A. thaliana* since the *Atelp2* mutant presents a delayed and reduced defense response. AtELP2 regulates cytosine methylation and histone acetylation levels on several defense genes, including several involved in responses to necrotrophic pathogens, such as *B. cinerea* and *A. brassicicola* [39, 40]. Research by Wang et al. showed that Elongator is required for full induction of the JA/ET defense pathway marker gene and for resistance to the necrotrophic fungal pathogens *B. cinerea* and *A. brassicicola*. The mutant with a loss-of-function mutation in the *A. thaliana* Elongator subunit 2 presents compromised resistance to the studied fungi, together with decreased histone acetylation and expression of JA/ET-defense genes *WRKY33*, *ORA59* and *PDF1.2* [41].

In the case of histone methylation, the situation is more complicated, because in addition to lysine residues, arginine residues can also be methylated in several places. Moreover, the matter is further complicated by the fact that specific methylation patterns are associated with both gene activation and repression [42]. In addition, there can occur mono-, di- and tri-methylation of histones, which present different physical properties. The strongest correlation between histone methylation and gene activity is found for trimethylation of Lys 4 on histone H3 (H3K4me3) on promoters and coding sequences of active genes [43]. H3K9me2, H3K27me3 and H2Aub1 are associated with transcriptional silencing/repression [44].

Many plant methylases and demethylases are involved in defense against pathogens. H3K36 methylation is involved in the regulation of PR gene transcription. In *A. thaliana*, set-domain group 8 (SDG8) is the main histone lysine methyltransferase catalyzing H3K36me2/3 [45]. SDG8 plays an important role in plant defense against fungal pathogens by modulating several genes in the JA and/or ET signaling pathways. Loss of SDG8 function causes mutant plants to show reduced resistance to the fungal pathogens *A. brassicicola* and *B. cinerea* [46]. In tomato, SDG8 orthologs were required for pathogen- and stress-induced enrichment of H3K36me3 and H3K4me3 at target genes, but their loss increased plant resistance to *B. cinerea* [47]. In addition to SDG8, SDG5 may also play role in plant immunity. Studies by Lee and co-authors indicate that in *A. thaliana*, SDG8 and SDG25 contribute to plant resistance to *B. cinerea* and *A. brassicicola*, either directly through histone lysine methylation or indirectly through H2B ubiquitination and by regulating plant resistance gene expression, lipid accumulation, carotenoid biosynthesis and maintaining integrity epidermis. However, SDG8 and SDG25 seem to have different molecular functions. SDG8 appears to perform the deposition of H3K36me2 and H3K36me3, while SDG25 may deposit H3K4me1 [48].

Histones can also be ubiquitinated on lysine residues, usually associated with transcriptional activation [44, 49]. *A. thaliana* RING E3 Histone Ligase Monoubiquitination 1 and 2 (HUB1 and HUB2) mediate the monoubiquitination of histone H2B. *A. thaliana* mutants deficient in *HUB1* showed higher sensitivity to *B. cinerea* and *A. brassicicola*, while overexpressing *HUB1* showed resistance to *B. cinerea*. The thickness of epidermal cell walls (acting as a physical barrier against invading fungal pathogens) is reduced in the *hub1* mutant [50]. *A thaliana* H2Bub is also involved in regulating the dynamics of microtubules during the defense response to toxins produced by the necrotrophic pathogen *Verticillium dahlia*, likely through the protein tyrosine phosphatase-mediated signaling pathway [51]. HUB1/HUB2 homologs in tomatoes, SlHUB1/SlHUB2, have similar H2B monoubiquitination E3 ligase activity, and their silencing leads to increased plant susceptibility to *B. cinerea* [52].

In rice, histone lysine 2-hydroxyisobutyrylation (Khib) is involved in regulation of PR gene expression. The ascomycete *Ustilaginoidea virens* may affect regulation of Khib in rice, where most of the Khib sites in histone H3 were downregulated during infection. RPD3-like histone deacetylase-HDA705 is involved in the removal of Khib in rice, negatively regulating plant resistance to *Ustilaginoidea virens*, while *HDA705* knockout increases resistance to these pathogens [53].

### Chromatin remodelers

**Fig. 3:**
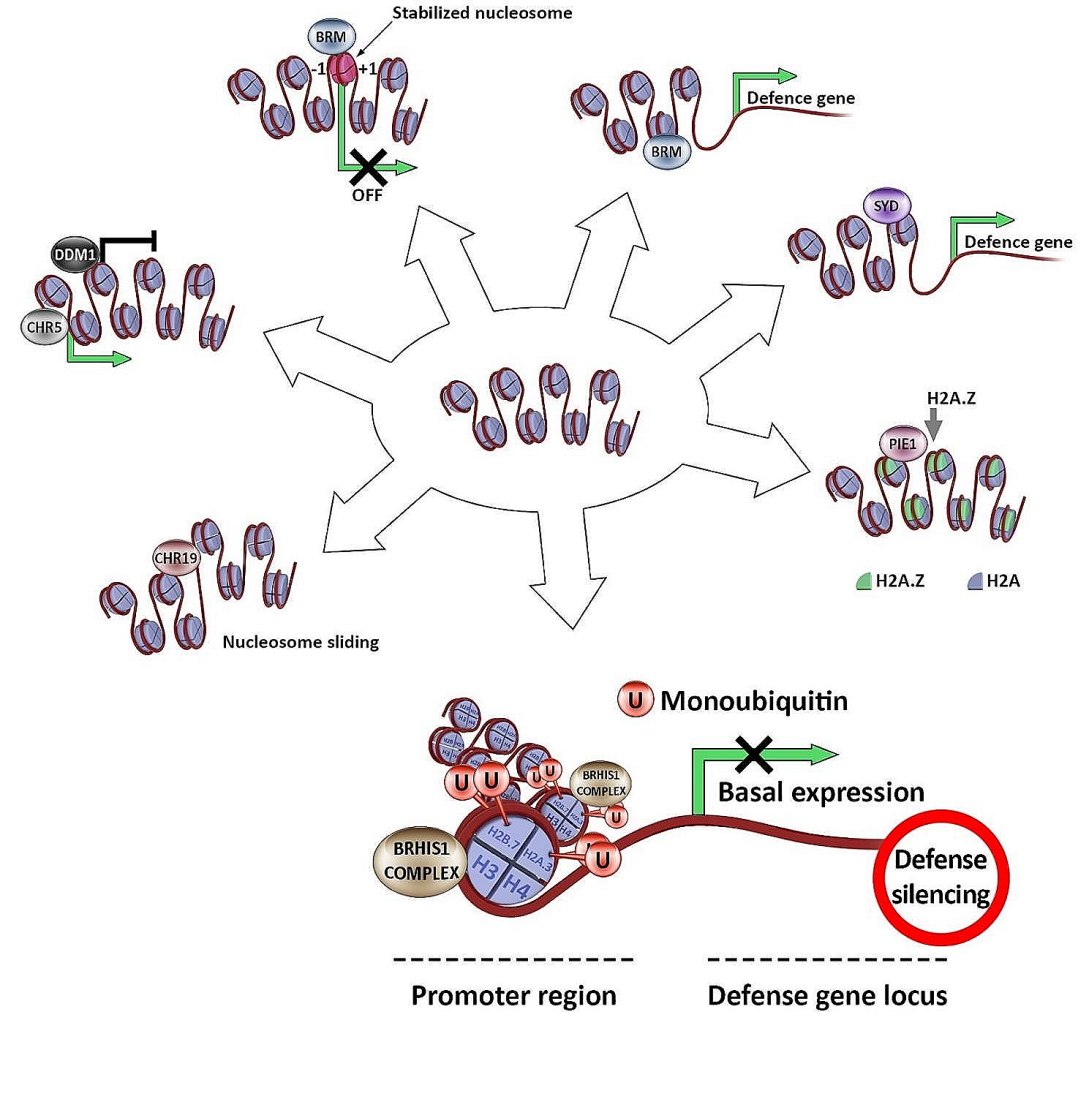
Possible mechanisms of ATP-dependent chromatin remodelers in the regulation of plant immunity during fungal infection. SYD (Splayed protein) and BRM (Brahma protein) are recruited to target loci and regulate the expression of defense-related genes. BRM also promotes the nucleosome stability. PIE1 (Photoperiod-Independent Early Flowering 1) is responsible for replacing the canonical histone H2A with a histone H2A variant. BRHIS1 (BIT-responsive Histone-interacting SNF2 ATPase 1) is recruited to the promoters of genes implicated in disease defense through interactions with monoubiquitinated histone variants. Under normal growth conditions, BRHIS1 binds to monoubiquitinated histones, suppressing the expression of disease defense-related genes. Upon pathogen attack, the down-regulation of BRHIS1 and the coincident up-regulation of H2A.Xa and H2B.7 displace BRHIS1 binding, resulting in gene activation. CHR19 (chromatin remodeler 19) is involved in nucleosome position mobilization (sliding). DDM1 (Decreased DNA Methylation 1) represses the transcription of plant defense genes during a pathogen attack. By contrast, CHR5 (chromatin remodeler 5) upregulates the transcription of plant defense genes

Chromatin remodeling not involving histone modifications is carried out by ATP-dependent chromosome remodeling complexes (CRC). Chromatin-remodeling factors can be divided into four subfamilies based on the catalytic Snf2 domain and other accessory domains: Switch/Sucrose on-fermentable (SWI/SNF), Inositol auxotrophy 80 (INO80), Imitation switch (ISWI), and Chromodomain helicase DNA-binding (CHD). In plants, members of these protein subfamilies have been associated with defense function [54] (Fig. 3).

Brahma (BRM) and Splayed (SYD) are the two most well-studied plant Snf2 proteins (belonging to the SWI/SNF subfamily). These proteins play roles in regulating plant developmental processes. SYD is required for expression of selected genes downstream of the jasmonate and ethylene signaling pathways. SYD is also directly recruited to the promoters of several of these genes. SYD contributes to defense against the necrotrophic pathogen *B. cinerea* in *A. thaliana* [55]. Research by Bezhani et al. indicated that *A. thaliana* SYD and BRM exhibit remarkable regulatory specificity by controlling the expression of a small number of targets involved in resistance [56].

Li et al. reported that the rice SWI/SNF2 ATPase gene *BRHIS1* (*BIT-responsive Histone-interacting SNF2 ATPase 1*) was downregulated in response to a rice fungal pathogen (*M. oryzae)* or to the defense-priming-inducing compound BIT (1,2-benzisothiazol-3(2 h)-one,1, 1-dioxide). Their results showed that rice disease defense genes are initially organized in an expression-ready state by specific monoubiquitination of H2A and H2B variants deposited on their promoter regions, but are kept suppressed by the BRHIS1 complex, facilitating the prompt initiation of innate immune responses in response to infection through the stringent regulation of BRHIS1. Inhibition of the expression of *BRHIS1* exhibited increased resistance to blast pathogens in rice, suggesting the negative regulatory role of BRHIS1 in plant immunity. After the plant receives a signal about the attacking pathogen, suppressed *BRHIS1* expression provokes the inaccessible chromatin to become accessible for defense gene expression through enhanced monoubiquitination of those targeted histone variants. The expression profile of defense genes in plants with suppressed *BRHIS1* expression indicates that there is a defense regulator that represses plant immunity in an SA-independent manner [57].

Another protein from the Snf2 family (INO80 subfamily) that may be involved in defense mechanisms is PIE1 (Photoperiod-Independent Early Flowering 1). PIE1 is part of the SWR1-like complex. One of the mechanisms involved in chromatin remodeling is the so-called ‘histone replacement’. An example of such a mechanism is the replacement of the canonical histone H2A with the variant histone H2A.Z, and in *A. thaliana* PIE1 is involved in this process. The H2A.Z variant interacts with PIE1. Loss of PIE1 function as well as depletion of H2A led to reduced basal resistance. There was a decrease in the level of expression of immunity genes; most of the mis-regulated genes were related to salicylic acid-dependent immunity. Functional knockout of these genes leads to constitutive activation of SA-mediated defense responses, and these mutants exhibit abnormal SA biosynthesis, overexpression of many SA-responsive genes, and spontaneous cell death under normal conditions, thus altering plant resistance to both biotrophic and necrotrophic pathogens. The SWR1-like complex is required for maintaining the repression of SA-dependent defense genes in unstressed plants [58, 59]. Cai et al. observed that the chromatin remodeling complex SWR1 enhanced resistance to the white mold fungus *Sclerotinia sclerotiorum* in *A. thaliana* via a process mediated by receptor-like kinase ERECTA (ER) signaling. The authors identified a series of WRKY33 target *YODA DOWNSTREAM* (*YDD*) genes and discovered that SWR1 and ER signaling was required to enrich H2A.Z histone variant and H3K4me3 histone modification at *YDD*s and the binding of WRKY33 to *YDD* promoters upon *S. sclerotiorum* infection. They also observed that the binding of WRKY33 to *YDD* promoters in turn promoted the enrichment of H2A.Z and H3K4me3 at *YDD* genes, thereby forming a positive regulatory loop to activate *YDD* expression. These results show the important role of chromatin structure in plant immune responses [60].

In *A. thaliana*, another ATPase of the INO80 subfamily, CHROMATIN REMODELER 19 (CHR19), has a conserved ATP-dependent nucleosome sliding activity. A variety of inducible genes, including several important genes in the salicylic acid and jasmonic acid pathways, were transcriptionally upregulated in the *chr19* mutant under normal growth conditions, indicative of a role of CHR19 in transcriptional repression. In addition, the *chr19* mutation triggered higher susceptibility to the JA pathway-defended necrotrophic fungal pathogen *B. cinerea*. CHR19 is involved in coordinating plant growth balance between development and stress response, and contributing to the improvement of plant resistance to fungal pathogens [61].

Decreased DNA Methylation 1 (DDM1) has a conserved SNF2 ATPase domain, which is required to maintain DNA methylation even though it has no methyltransferase activity itself [62] DDM1 controls R genes. DDM1 regulates expression of the *A. thaliana* plant resistance gene *SNC1 (suppressor of npr1-1*, *constitutive1*; one of the R genes) antagonistically to MOS1 (modifiers of snc1), by regulating the methylation levels in the upstream region of the gene and influencing the level of resistance to *Hyaloperonospora arabidopsidis* [63]. The repression of *SNC1* expression in *mos1 snc1* mutant plants of *A. thaliana* can be released by knocking out *DDM1*. It is thus likely that the repression of *snc1* expression in *mos1 snc1* is caused by altered chromatin structure rather than changes in DNA methylation. CHR5 (Chromatin-Remodeling Factor 5) acts as an antagonist to DDM1 in the regulation of SNC1. CHR5 also functions independently with the histone mono-ubiquitinase HUB1 in SNC1 regulation [64].

### DNA methylation

**Fig. 4:**
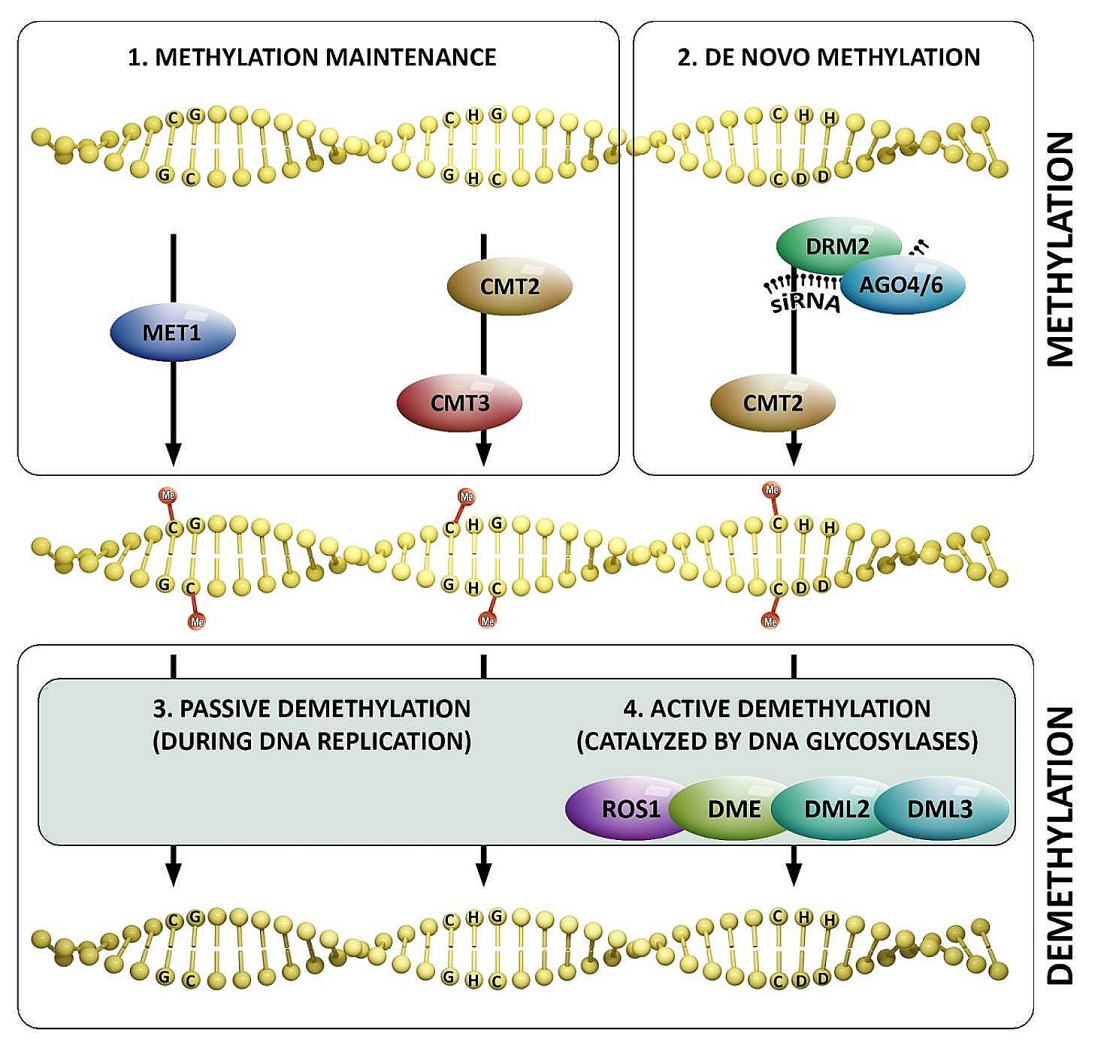
DNA methylation and demethylation in plants. Methylation in plants can occur through two processes: DNA methylation maintenance [1] and de novo DNA methylation [2]. Methyltransferase 1 (MET1) maintains symmetric CG site methylation. Chromomethylase 2 and 3(CMT 2, CMT 3) maintains symmetrical CHG site methylation. De novo CHH methylation is performed by domain-rearranged methyltransferase 2 (DRM2) or CMT2. DRM2 induces CHH methylation via the RNA-directed DNA methylation (RdDM) pathway, relying on the presence of 24 nt small interfering RNA (siRNA). This siRNA is loaded onto ARGONAUTE proteins (AGO), primarily AGO4 and AGO6, which then interact with DRM2. Demethylation of DNA encompasses passive demethylation [3] and active demethylation [4]. Replacement of 5mC with unmethylated cytosine during passive demethylation involves the binding of nuclear factor (NF) to 5mC during DNA replication. This binding makes it difficult to maintain DNA methylation, causing loss of DNA methylation on the newly synthesized strand. Active demethylation relies on the removal of 5mC by DNA glycosylases: repressor of silencing 1 (ROS1), Demeter (DME), Demeter-like 2 and 3 (DML2, DML3). These DNA glycosylases can remove 5-mC from any sequence context

DNA methylation and demethylation affect the resistance of plants to fungal diseases, probably due to the regulation of many genes of the immune response. DNA cytosine methylation is one of the major epigenetic mechanisms in higher eukaryotes and plays a key role in maintaining genome stability and regulating gene expression. DNA methylation targets tandem and interspersed repeats. At these loci, DNA methylation occurs in three different sequence contexts: symmetrical CG dinucleotides; symmetrical CHG, where H corresponds to A, T, or C; and asymmetrical. DNA methylation can also be found in gene bodies, exclusively at CG residues [65]. Cytosine methylation is established in all sequence contexts by de novo methyltransferases (DRM1/2) via the small interfering RNAs (RdDM) DNA methylation pathway. Here, DICER-dependent 21- to 24-nt siRNAs direct Argonaute proteins (AGO4/AGO6) to complementary sequences in the genome, possibly through a base-pairing mechanism and siRNA:nascent RNA base pairing mechanism to guide cytosine methylation. CG and CHG methylation is maintained through DNA replication by MET1, a homolog of the mammalian DNA methyltransferase DNMT1, and the plant-specific CMT3 methyltransferase. Active demethylation of methylcytosines is catalyzed by the DEMETER (DME) family of DNA glycosylases, while passive demethylation of methylcytosines is a consequence of DNA replication [65, 66] (Fig. 4).

RNA-directed DNA methylation (RdDM) is an epigenetic control mechanism driven by small interfering RNAs (siRNAs) that influence gene function. *NRPD2* encodes the second largest subunit of the plant-specific RNA Polymerases IV and V (Pol IV and Pol V), which are crucial for the RdDM pathway. NRPD2 is the Overexpressor of Cationic Peroxidase 1. Lopez et al. evaluated the resistance of effective RdDM-deficient mutants *of A. thaliana*. Mutants of *nrpe1*, *nrpd2*, *rdr2*, *drd1*, *ago4-2*, and *drm1drm2*, except for *nrpd1*, exhibited increased susceptibility to *B. cinerea*. The results indicated that the RdDM pathway positively regulates resistance against necrotrophic fungi, presumably by promoting JA signaling during infection. The authors also suggested that while the Pol V complex is required for plant resistance, Pol IV appears to be redundant [67].

*A. thaliana* encodes four DNA demethylases: DEMETER (DME), Repressor Of Silencing 1 (ROS1), DEMETER-Like 2 (DML2), and DML3. Le et al. reported that ROS1, DML2 and DML3 play a role in fungal disease resistance in *A. thaliana*. A triple DNA demethylase mutant, *rdd* (*ros1 dml2 dml3*) shows increased susceptibility to the fungal pathogen *Fusarium oxysporum*. The expression of a significant number of genes involved in plant resistance was downregulated in mutants, suggesting that DNA demethylases maintain or positively regulate the expression of stress response genes required for *F. oxysporum* resistance. Stress response genes with downregulated rdd are enriched with short sequences of transposable elements in their promoters. Many of these transposable elements and their surrounding sequences show localized changes in DNA methylation in rdd and an overall reduction in CHH methylation, suggesting that the RNA-directed DNA methylation responsible for CHH methylation may be involved in DNA demethylase-mediated regulation of stress response genes. The results obtained by the authors also suggest that DNA demethylases target short promoter TEs (transposable elements) sequences to regulate these stress response genes. It is possible that the products of these genes, required for the stress response, are detrimental to plant development if accumulated at high levels and therefore must be suppressed under normal growth conditions via promoter methylation. Under stress conditions, these genes are temporarily activated by the action of DNA demethylases [68]. Many types of TEs have been identified in R-gene clusters. TE activity is usually suppressed by methylation or expressed at low levels as a result of methylation along their entire length [69]. TE methylation sometimes spreads into the genic regions of flanking genes, and suppresses their transcription [70]. Co-expression of genes and TEs is possible, in particular for long terminal repeat (LTR) elements. For example, the rice LTR retrotransposon Renovator is present in the promoter region of the *Pit* resistance gene in specific rice cultivars. The functional allele of PitK59 contains four amino acid substitutions and has the LTR retrotransposon Renovator inserted upstream, which enables expression of the rice blast resistance gene and confers resistance to *Magnaporthe grisea*. The 5′ side of Renovator is heavily methylated, whereas the 3′ side is only partially methylated. LTR methylation status correlates with transcriptional activity, suggesting that hypomethylation of the 3′ side of the Renovator sequence may be needed for higher *Pit* expression [71]. The above-mentioned and other examples confirming the role of DNA methylation in the response of plants to infections with pathogenic fungi are presented in Table 1.

**Table 1:** Involvement of DNA methylation in the response of plants to infections caused by pathogenic fungi

DNA methylation	Plant / pathogen	Influence on immunity	Ref.
RdDM pathway positively regulates resistance against necrotrophic fungi presumably by promoting JA signaling	*A. thaliana* */* *B. cinerea*	positive	[67]
ROS1, DML2, and DML3 maintain or positively regulate expression of stress response genes	*A. thaliana* */* *F. oxysporum*	positive	[68]
Hypomethylation of the LTR region in the promoter of the rice blast resistance gene *Pit* may be needed for higher *Pit* expression	*Oryza sativa* / *Magnaporthe grisea*	positive	[71]
Silencing MET1 (MET1 maintains the CG methylation of resistance genes)	Mulberry (*Morus notabilis*) / *B. cinerea*	positive	[72]
Most of the promoters of defense genes were hyper-methylated	Canola (*Brassica napus*) / *Leptosphaeria maculans*	positive	[73]
Mutants with downregulated *DME* showed increased susceptibility to fungal pathogens	*A. thaliana* / *V. dahliae*	negative	[74]
Change in methylation levels in multiple regions, especially in transposable element regions, during genome-wide analysis; for most regions, predominance of hypermethylation	Rice (*Oryza sativa* L. ssp. Indica) / *M. oryzae*	negative	[75]
In control and infected plants 932 differentially expressed genes (a set of resistance-related genes including *R* genes and candidate genes in metabolic and defense pathways) were associated with hypermethylation, and 603 with hypomethylation	Melon (*Cucumis melo* L.) / *Podosphaera xanthii*	positive	[76]
High levels of methylation at the *DFR* and *RUBY* promoters (genes involved in the anthocyanin biosynthetic pathway)	Blood Orange (*Citrus sinensis* L. (Osbeck)) / *Penicillium digitatum*	positive	[77]
Differentially methylated regions with CHH-hypomethylated	*Aegilops tauschii* / *Blumeria graminis* f. sp. *tritici* (Bgt)	positive	[78]

### Non-coding RNA

**Fig. 5:**
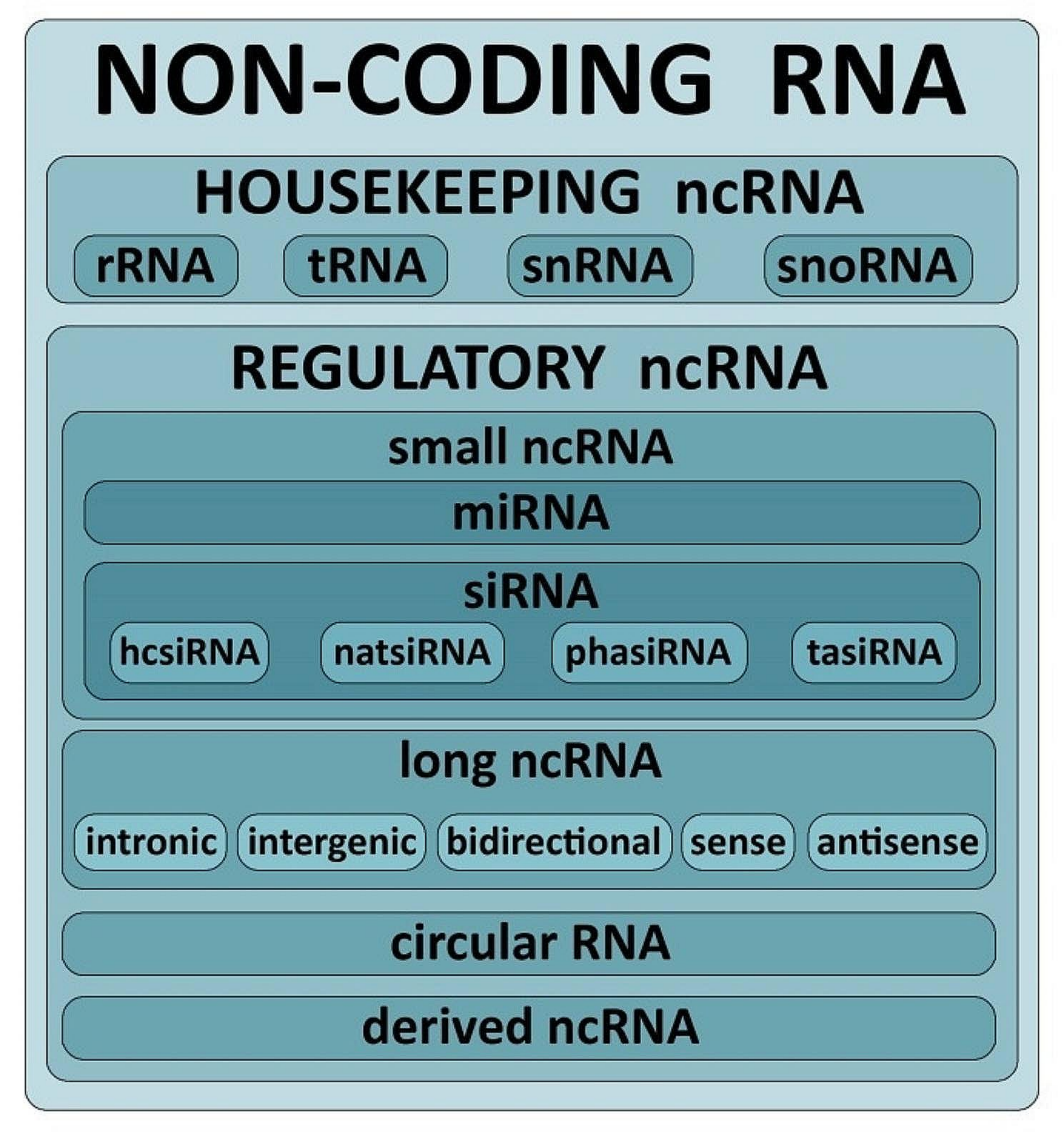
Classification of non-coding RNAs in plant. Non-coding RNAs are classified into housekeeping and regulatory ncRNAs based on their respective roles in biology. Regulatory ncRNAs are further divided into classes and subclasses based on their nucleotide length, structure, and function. Abbreviations: ncRNA, non-coding RNA; tRNA, transfer RNA; rRNA, ribosomal RNA; snRNA, small nuclear RNA; snoRNA, small nucleolar RNA; miRNA, micro RNA; siRNA, small interfering RNA; hcsiRNa, heterochromatic small interfering RNAs; phasiRNAs, phased secondary small interfering RNAs, tasiRNAs- trans-acting small interfering RNAs, natsiRNA, natural antisense short interfering RNA

In addition to the role of protein-coding genes as key regulators of plant immunity, increasing evidence indicates the importance of non-coding RNAs (ncRNAs) in plant immune responses. Depending on their mode of biogenesis and their functions, ncRNAs have evolved into various forms that include microRNAs (miRNAs), small interfering RNAs (siRNAs), lncRNAs, circular RNAs (circRNAs), and derived ncRNAs (Fig. 5). NcRNAs may act as epigenetic modulators through chromatin remodeling or regulate gene expression at the transcriptional or posttranscriptional level [79, 80].

One of gene regulatory mechanism is RNA silencing. This process involves sRNA. sRNAs are divided into two major classes: miRNAs and siRNAs. miRNAs are usually 21–24 nucleotides (nt) long and are derived from RNAs with imperfectly base-paired hairpin structures. siRNAs are generated from perfectly complementary long double-stranded RNAs (dsRNAs) and may require RNA-dependent RNA polymerases [81].

One of the first reported miRNAs involved in plant resistance was miR393 from *A. thaliana*. It was induced by a PAMP, flg22, and silenced auxin receptors to turn down the auxin signaling pathway, resulting in activation of PTI [82]. Cassava anthracnose disease is caused by the fungus *Colletotrichum gloeosporioides*, and can cause tip die-backs and stem cankers. Quantitative RT-PCR analysis revealed differential expression of the two miRNAs and their target genes in the two cassava cultivars that had been subjected to fungal infection. The more resistant variant revealed upregulation of miR160 and miR393, which consequently led to low levels of ARF10 and TIR1 transcripts, respectively. The more susceptible variety showed the opposite pattern. Cis-regulatory elements relevant to defense and stress responses, fungal elicitor responses, and hormonal responses were most prevalent in the promoter regions of miRNA genes [83]. In *A. thaliana* miR396 levels gradually decreased during fungal infection, thus enabling its growth-regulating factor (GRF) transcription factor target genes to trigger host reprogramming, enabling defense against *Plectosphaerella cucumerina*, a destructive necrotrophic fungal pathogen [84].

Comparison using deep sequencing of small RNA libraries from the resistant and sensitive variety* O. sativa* allowed Li et al. to identify a group of miRNAs that were differentially expressed after infection with *M. oryzae*. Moreover, transgenic rice plants overexpressing miR160a and miR398b displayed enhanced resistance to *M. oryzae*, as demonstrated by decreased fungal growth, increased hydrogen peroxide accumulation at the infection site, and up-regulated expression of defense-related genes. miR398b overexpression reduced the transcript levels of genes encoding superoxide dismutases (CSD1, CSD2, SODX, and CCSD), leading to elevated ROS production and enhanced plant resistance against *M. oryzae* [85, 86]. Another example of non-coding RNAs involved in the regulation of ROS production in response to fungal infection is miR400 in *A. thaliana*. This miRNA directs the cleavage of two pentatricopeptide repeat genes, resulting in increased ROS accumulation and impaired resistance to *B. cinerea* [87]. In *A. thaliana* interference with miR773 activity by target mimics (in MIM773 plants) and concomitant upregulation of the miR773 target gene methyltransferase 2 (MET2) increased resistance to infection by necrotrophic (*Plectosphaerella cucumerina*) and hemibiotrophic (*F. oxysporum*, *Colletotrichum higginsianum*) fungal pathogens. Upon pathogen challenge, these plants accumulated higher levels of callose and reactive oxygen species than wild-type plants [88].

Silencing of individual cDNA clones in wheat challenged with *Puccinia striiformis* f. sp. *tritici* indicated that the chemocyanin-like protein gene *TaCLP1* (the target gene of wheat miRNA R408) positively regulated resistance to stripe rust [89]. After infection of barley with the fungus *Blumeria graminis* f. sp. *hordei*, there was significant reprogramming of gene expression mediated by Mla- Mildew resistance locus a. Xu et al. utilized a proteomics-based approach, combined with barley *Mla*, required for *Mla12 resistance1* (*rar1*), and restoration of *Mla resistance1* (*rom1*) mutants, to identify components of *Mla*-directed signaling. Loss-of-function mutations in *Mla* and *Rar1* both resulted in the reduced accumulation of chloroplast copper/zinc superoxide dismutase 1 (HvSOD1), whereas loss of function in *Rom1* re-established HvSOD1 levels. *Mla* and *Rom* repress miR398-mediated SOD1 expression to change the hypersensitive reaction response to fungus [90]. Campo et al. investigated miRNAs that are regulated by elicitors from the blast fungus *M. oryzae* in rice (*O. sativa*). Elicitor treatment was accompanied by dynamic alterations in the expression of a significant number of miRNAs. They reported a rice miRNA, osa-miR7695, which negatively regulates an alternatively spliced transcript of OsNramp6 (Natural resistance-associated macrophage protein 6). Overexpression of this miRNA in rice confers pathogen resistance [91]. Li et al. found that miR169 acts as a negative regulator in rice immunity against the blast fungus *M. oryzae* by repressing the expression of *nuclear factor Y-A* (*NF-YA*) genes [92]. Another miRNA – osa-miR164a – is also involved in the defense of rice against this fungus. Wang et al. found that the expression of osa-miR164a decreased after infection with *M. oryzae* in both early and late stages, which was associated with induced expression of its target gene, *OsNAC60*, which encodes a transcription factor whose overexpression enhances defensive responses such as increased programmed cell death, greater ion leakage, greater accumulation of reactive oxygen species and callose deposition, and upregulation of defense-related genes. In transgenic rice where the miR164a/*OsNAC60* regulatory module was dysfunctional, plants developed significant susceptibility to infection by *M. oryzae* [93].

Rice plants with activated, conserved, polycistronic, miR166 expression showed enhanced resistance to infection by the fungal pathogens *M. oryzae* and *Fusarium fujikuroi*. Disease resistance was associated with stronger expression of defense responses during pathogen infection. miR166k positively regulates rice immunity by controlling expression of the *EIN2* (*ethylene-insensitive 2*) gene, which encodes a critical regulator of ethylene signaling [93].

Several miRNA families target genes encoding plant innate immunity receptors with a leucine-rich nucleotide binding site (NBS-LRR). Cotton plants can induce expression of NBS-LRR defense genes by suppressing the miRNA-mediated gene silencing pathway (miR482) after attack by the fungal pathogen *V. dahlia* [94]. Ouyang et al. investigated the role of miRNAs in tomato defense against *F. oxysporum* using comparative miRNA profiling of susceptible (Moneymaker) and resistant (Motelle) tomato cultivars. The authors focused on two miRNAs – slmiR482f and slmiR5300 – which are exceptionally downregulated in the resistant variety during fungal infection. Two predicted mRNA targets each of slmiR482f and slmiR5300 exhibited increased expression in Motelle. All predicted targets of these miRNAs encode proteins with nucleotide-binding (NB) domains and a motif associated with plant pathogen resistance. None of these targets correspond to I-2, the only known resistance (R) gene for *F. oxysporum* in tomatoes, which supports roles for additional R genes in the immune response. Fungus infection down-regulates the accumulation of miRNA to increase the expression of NB domain genes [95].

Analysis by Ma et al. showed that one of the miRNAs, Md-miRLn11 (*Malus domestica* microRNA Ln11), targeted an apple nucleotide-binding site (NBS)-leucine-rich repeat (LRR) class protein coding gene (*Md-NBS*). Analysis of the expression of *Md-miRLn11* and *Md-NBS* during the optimum invasion period in 40 apple varieties showed that expression of the *Md-NBS* gene in resistant varieties is higher than in susceptible varieties, with an inverse pattern for Md-miRLn11. Infection with the fungus-causing apple leaf blotch on a seedling of a resistant apple cultivar showed a marked decrease in apple resistance. On the other hand, a susceptible apple cultivar with infiltration of the *Md-NBS* gene showed a significant increase in disease resistance [96]. In *Brassica napus*, 41 lncRNAs were identified as precursors for microRNAs, including miR156, miR169 and miR394, with significant roles in mediating plant responses to the fungal phytopathogen *Sclerotinia sclerotiorum* [97]. The abovementioned and other examples confirming the participation of miRNA in the plant defense response against infection with pathogenic fungi are presented in Table 2.

**Table 2:** Involvement of miRNA in the response of plants to infections caused by pathogenic fungi

miRNA /target	Plant / pathogen	Influence on immunity	Ref.
miR160, miR393	*Cassava (Manihot esculenta Crantz)* */* *C. gloeosporioides*	positive	[83]
miR396	*A. thaliana* / *P. cucumerina*	negative	[84]
miR160a, miR398b	*Rice (O. sativa)/M. oryzae*,	positive	[85]
miR398b boosts total SOD activity improve disease resistance.	Rice (*Oryza sativa*) */ M. oryzae*;	positive	[86]
miR400	*A. thaliana* */* *B. cinerea*	negative	[87]
miR773	*A. thaliana* */* *P. cucumerina, F. oxysporum, C. higginsianum*	negative	[88]
miRNA R408	*Triticum aestivum /Puccinia striiformis* f. sp. *tritici*	positive	[89]
miR398	*Hordeum vulgare L.* */* *Blumeria graminis*	negative	[90]
miR7695	Rice (*O. sativa)/M. oryzae*	negative	[91]
miR169	Rice (*O. sativa)/M. oryzae*	negative	[92]
miR146	Rice (*O. sativa)/M. oryzae*	negative	[93]
miR482	*Cotton (G. hirsutum G. arboretum)* */* *V. dahliae*	positive	[94]
miR482f, miR5300,	*Tomato (cv.)* Motelle *and* Moneymaker */* *F. oxysporum* f. sp. *lycopersici*	negative	[95]
miRLn11	Apple (*M. domestica* cv. Golden Delicious)	negative	[96]
miR319a */* BraTCP4 ((TEOSINTE BRANCHED 1 */* CYCLOIDEA/PCF) involved in JA biosynthesis and regulators of plant growth))	*B. rapa* */* *S. sclerotiorum*	negative	[98]
miR159, miR5139, and miR390, miR1885	*B. napus* */* *S. sclerotiorum*	positive	[99]
miR6024/NLR genes	Tomato (*Solanum lycopersicum* L.) cultivar Pusa Ruby */* *Alternaria solani*	negative	[100]
miR319c/TCP29.	Tomato (*S. lycopersicum*) cv. MicroTom */* *B. cinerea*	positive	[101]
miR482b	Tomato *cv. MicroTom* */* *B. cinerea*	negative	[102]
miR1127-3p / WRKY75	Tomato (*Solanum lycopersicum* cv. Ailsa Craig) */* *B. cinerea* strain B05.10	positive (miRNA repression)	[30]
up-regulation of GhmiR395 */* APS1/3, adenosine 5′-phosphosulfate; down-regulation of GhmiR165 */* REV- transcription factors	Cotton (*Gossypium hirsutum L.)* */* *V. dahliae*	positive	[103]
miR398b */* *NBS-LRR* gene and cleavage of the mRNAs of superoxide dismutase and Copper chaperone for superoxide dismutase	Cotton (*G. hirsutum* cv. and *G. barbadense)* */* *V. dahliae*	negative	[104]
miR530 */* Stress-associated protein 6 gene	Cotton (*Gossypium hirsutum* L.) */* * V. dahliae*	silencing of miR530 – positive, while its overexpression – negative.	[105]
miR477/CBP60A (CaM-binding protein 60)	Cotton (*Gossypium hirsutum* L.) */* * V. dahliae*	negative – (miR477 silencing)	[106]
ghr-miR164 */* NAC100 (plant-specific transcription factors)	Cotton (*Gossypium hirsutum* L.) */* * V. dahliae*	positive	[107]
miR482b, miRN2031, -miR398 /resistance proteins gene	*Nicotiana benthamiana* */* *Alternaria longipes*	positive	[108]
zma-unmiR4 */* *ZmGA2ox4* gene- encoding gibberellin 2-oxidase 4	Maize susceptibility and resistance lines */* *Fusarium verticillioides*	negative	[109]
overexpressing miR408b	Maize susceptibility and resistance lines */* *Fusarium verticillioides* (FER)	negative	[110]
miR-156, miR167, miR171, miR408, miR444 */* SBP*-*Box transcription factor *SPL3;* MADS-box transcription factor	Rice (*O. sativa*) */* *Rhizoctonia solani*	positive	[111]
miR319a/ *TCP10*	Tea plant (*Camellia sinensis*) */* *Pestalotiopsis*	negative	[112]
miR477 */* *phenylalanine ammonia-lyase* gene	Tea plant (*C. sinensis*) */* *Pseudopestalotiopsis*	negative	[113]
miR156/SPL9 (squamosa promoter binding protein-like 9)	*A. thaliana* */* *B. cinerea*	positive	[114]
GSH-responsive miRNAs */* defense-related genes like leucine-rich repeat protein kinase, MYB transcription factors, TCP8	*A. thaliana* */* *A. brassicicola*	positive	[115]
certain miRNA/targets of the miRNAs include transcription factors, membrane-bound proteins, glutamate receptor proteins, lignin biosynthesis proteins, signaling cascade proteins, transporter proteins, mitochondrial proteins, ER proteins, defense-related, stress response proteins, translational regulation proteins, cell proliferation, and ubiquitination proteins. miR444b.2 */* *OP1,TSMBP*, *CF_9, NSSTPK* miR444a/*COEPP16*, *STPK-PB51*, *ELGNBP3*, *LANT* miR5568b */* *EMB2745*, *PCRBP*, *SBP*, *NSSTPK1* miR169b/*CAD7*, *OSJP*, *CTPT* miR166d.5p/*PARN*, *OSP1*, *OSPL*, *BPH14*, *RP-RGA4*, *RLP*, *PM-CTA-10* miR164c */* *DLN2*, *GBPE* miR162b */* *CHP*, *FLXL3*	Sugarcane */* *Colletotrichum falcatum*	positive negative	[116]
Osa-miR444b.2 */* affecting the expression of plant hormone signaling pathways-related genes such as ET and IAA, and transcription factors such as WRKYs and F-boxes.	Rice (*O. sativa* L *)* */* *Rhizoctonia solani*	negative	[117]
miRcand137 */* ERF14, encoding a transcription activator of the ERF family that participate in defense-related pathways.	Apple tree (*M. domestica*) */* *Botryosphaeria dothidea*	negative	[118]
miRNA397 */* LAC7 (involved in lignin biosynthesis)	Apple tree (*M. hupehensis)* */* *Botryosphaeria dothidea*	negative	[119]

Gene expression mediated by siRNA plays an important role in regulation of plant growth, development and immunity. For example, Qiao et al. characterized the Tourist-miniature inverted-repeat transposable element (MITE)-derived siR109944 as having a conserved function that enhanced susceptibility to *Rhizoctonia solani* infection by affecting auxin homeostasis in rice and *A. thaliana*. One potential target of siR109944 is the F-Box domain and LRR-containing protein 55, which encode the transport inhibitor response 1 (TIR1)-like protein. The authors found that rice had significantly enhanced *R. solani* susceptibility when siR109944 was overexpressed. Overexpression of siR109944 in *A. thaliana* also increased susceptibility to *B. cinerea*, *Sclerotinia sclerotium*, and *V. dahliae* infection [120, 121]. In rice, the majority of characterized sRNAs are within the range of 21 to 24 nt long. Niu et al., using deep sequencing on rice plants, identified a group of small interfering RNAs between 25 and 40 nt in length, which constitute another class of sRNA. The results indicate that some rice this group of siRNA are differentially regulated after infection with the fungal pathogen *R. solani*, which causes rice sheath blight disease, and may also target genes related to defense [122].

Plants contain a large number of intracellular nucleotide-binding NLR immune receptors, encoded by resistance (R) genes, which recognize specific pathogen effectors and trigger resistance responses. Disease-related genes, particularly the NLR class of R plant genes, can be triggered by miRNAs to generate phasiRNAs (phased small interfering RNA), which could reduce the transcript levels of their targets. phasiRNAs are phased, secondary, small interfering RNAs, mostly triggered by the cleavage of the target mRNAs by 22-nucleotide (nt) miRNAs. phasiRNAs are widely present in plants, and they can regulate their target genes by cleaving mRNAs at the post-transcriptional level or directing DNA methylation at the transcriptional level. phasiRNAs can suppress the expression of NLR genes when there is no pathogen. Upon pathogen infection, the phasiRNAs are generally downregulated, which leads to upregulation of NLRs and activation of immune responses [123, 124]. For example, Liu et al. observed in barley that 22-nt miR9863 triggered 21-nt phasiRNA biogenesis and together repressed the expression of group I *Mla* alleles. Barley *Mla* alleles encode coiled-coil (CC), nucleotide binding, and NLR receptors that trigger isolate-specific immune responses against the powdery mildew fungus, *Blumeria graminis* f. sp. *hordei* [125]. Hunt et al. also identified barley phasiRNAs in response to Bgh infection, which overlap transcripts that encode receptor-like kinases (RLKs) and nucleotide-binding, leucine-rich domain proteins (NLRs). These include phasiRNA loci that overlap with a significant proportion of receptor-like kinases, suggesting that an additional sRNA control mechanism may be active in barley leaves as opposed to predominant R-gene phasiRNA overlap in many eudicots [126].

The expression of some NLRs is also regulated by transcriptional silencing by heterochromatic small interfering RNAs (hc-siRNAs) produced in the RDR2-DCL3-AGO4 pathway by plant-specific RNA polymerase IV and V. hc-siRNAs are usually 24–30 nt in length, derived mainly from transposons and repeats, and direct de novo DNA methylation and histone modifications at their target sites [127]. An example is the Rice *Pygm* locus, which confers lasting resistance to the fungus *M. oryzae*. *PigmS* expression and thus PigmR-mediated resistance are subjected to tight epigenetic regulation. The *PigmS* promoter contains two tandem miniature transposons, which associate with hc-siRNAs [128].

Not only short non-coding RNAs play an important role in the regulation of gene expression and response to biotic stress, but also long non-coding RNAs (lncRNAs) may perform such a function. lncRNAs are transcribed from a variety of genomic locations (introns, intergenic spaces, and coding regions) from the sense or antisense strand. lncRNAs function in cis or in trans and affect gene regulation transcriptionally or posttranscriptionally by diverse mechanisms, including recruiting factors that activate transcription or modify chromatin, serving as precursors of small RNAs, and even potentially affecting nuclear architecture. Plant lncRNAs function in RNA-directed DNA methylation (via production of small interfering RNAs) [129]. Bhatia et al., using a computational approach based on RNA-seq data, identified 71 powdery mildew-responsive *Vitis vinifera* lncRNAs. Further analysis revealed lncRNA association with Ca^2+^-binding proteins such as calmodulin/calmodulin-like proteins, enzymes involved in reactive oxygen species (ROS) metabolism, cell-wall modification/reinforcement, secondary metabolic pathways, phytoalexin (like resveratrol) production, pathogenesis-related proteins such as PR-1, PR-4 and PR-10, and phytohormone-based signal transduction [130]. Comparison of melon varieties sensitive and resistant to this pathogen also confirmed the role of lncRNA in plant defense against fungi [131].

With a strand-specific RNA-sequencing approach, Zhu et al. detected several lncRNAs induced in *A. thaliana* in response to infection with the fungus *F oxysporum*. Several noncoding natural antisense transcripts responsive and 20 responsive long noncoding transcriptionally active regions (lncTARs) to *F. oxysporum* infection were found in genes implicated in disease defense. Ten *F. oxysporum*-induced lncTARs were functionally characterized using T-DNA insertion or RNA-interference knockdown lines, and five were demonstrated to be related to disease development. Knockout mutants for these *lncTARs* presented higher susceptibility to this fungus, indicating their role in the regulation of defense. Regulation of the *At2g30770* gene and its long noncoding natural antisense transcripts (*lncNATs*) was coordinated in response to *F. oxysporum* infection. The *At2g30770* gene contains a TCA-element responsive to salicylic acid, and a TC-rich repeat responsive to stresses in its core promoter. Additionally, promoter analyses suggested that some of the *F. oxysporum*-induced lncTARs are direct targets of transcription factor(s) (TF) responsive to pathogen attack. Sense transcripts involved in the defense response and their lncNATs could be expressed in the same direction, which could be dependent or independent owing to the presence of similar TF binding sites upstream and downstream of protein-coding genes or similar pathogen-responsive elements in their promoter [132]. Sense and antisense strands were transcribed in the opposite direction and a negative correlation was observed between sense and antisense strand expression in both susceptible and resistant wheat cultivars, in response to powdery mildew infection. The comparison of lncRNA expression profiles between wheat species sensitive and resistant to *Blumeria graminis* f. sp. *tritici* exposed to this pathogen revealed that numerous lncRNAs were differentially expressed. Among them, some were precursors of small RNAs such as microRNAs and siRNAs, two long non-protein-coding RNAs (npcRNAs) were identified as signal recognition particle (SRP) 7 S RNA variants, and three were characterized as U3 small nucleolar RNAs (snoRNAs). In addition, lncRNAs were expressed in a tissue-specific manner [133]. In mulberry, the lncRNA *MuLnc1* was found to be cleaved by mulmiR3954. It was observed that one of the siRNAs produced, si161579, could silence the expression of the *calmodulin-like protein* gene *CML27* of mulberry (*MuCML27*). When *MuCML27* was heterologously expressed in *A. thaliana*, the transgenic plants exhibited enhanced resistance to *B. cinerea* [134]. The most commonly cultivated grapevine species – *V. vinifera* – is susceptible to many pathogens, one of which is *B. cinerea*. The results obtained by Bhatia et al. indicate that lncRNAs, along with other regulators such as miRNAs, help modulate the basic plant defense response to necrotrophic fungi. The lncRNAs identified by the authors may coordinate in a grapevine defense response to *B. cinerea* that includes fungal chitin degradation, stilbenoid accumulation, ROS detoxification, and cell wall reinforcement [135]. Other research conducted on vines infected with *Lasiodiplodia theobromae* also confirmed the role of lncRNA in defense against fungal pathogens by affecting the target genes involved in cell wall organization and chitin signaling [136]. Zhau et al. identified 539 lncRNAs from powdery mildew-resistant (MR-1) and susceptible melon (Top Mark), of which 254 were significantly altered after fungal infection. These lncRNAs might be involved in the hydrolysis of chitin, callose deposition, plant-pathogen interaction pathway and the plant hormone signal transduction pathway [137]. The above-mentioned and other examples confirming the involvement of lncRNA in the plant defense response to fungal infections are presented in Table 3.

**Table 3:** Involvement of lncRNA in the response of plants to infections caused by pathogenic fungi

lncRNA /target	Plant / pathogen	Influence on immunity	Ref.
identified 71 different, potential powdery mildew-responsive lncRNAs */* PR-1, PR-4 and PR 10 proteins, Ca2+-binding proteins, ROS enzymes involved in reactive oxygen species (ROS) enzymes involved in cell-wall modification, secondary metabolic pathways, signal transduction	V. vinifera */* *E. necator*	positive	[130]
407 potential lncRNAs in susceptible melons and and 611 lncRNAs in resistant melons in response to infection */* 1232 different target	Melon (*Cucumis melo L.*) */* Powdery mildew fungus	-	[131]
lncTARs	*A. thaliana* */* *F. oxysporum*	positive	[132]
npcRNAs	Wheat (*T. aestivum* */* *Blumeria graminis* f. sp. *tritici*	negative and positive	[133]
MuLnc1 */* *MuCML27*	Mulberry (*Morus* spp.); transgenic *Arabidopsis* with heterologously expressed *MuCML27/B*. cinerea	positive	[134]
826 lncRNAs, lincRNAs and lncNATs	Grapevine */* *Lasiodiplodia theobromae*.	negative and positive	[136]
254 lncRNAs were significantly altered after infection */* target genes involved in the hydrolysis of chitin, callose deposition and cell wall thickening, plant-pathogen interaction and plant hormone signal transduction pathway	Melon (*C. melo* L.) */* powdery mildew	negative and positive	[137]
lncLOX3 */* GhLOX3 implicated in JA biosynthesis	Cotton (*Gossypium hirsutum*) */* *V. dahliae*	negative (lncLOX3-silenced)	[138]
lncRNA7 */* *Pectin methylesterase inhibitor 13* gene (*GbPMEI13*)	Cotton CSSLs were developed by backcrossing *G. hirsutum* and *G. barbadense* */* *Verticillium wilt*	positive	[139]
lncRNA2 */* *Polygalacturonase 12* (*GbPG12*) gene	Cotton CSSLs were developed by backcrossing *G. hirsutum* and *G. barbadense* */* * V. wilt*	negative	[139]
In total 83 LncRNAs were up-regulated after blast fungus infection and 78 were down-regulated. One up-regulated lncRNA was derived from a jasmonate (JA) biosynthetic gene, *lipoxygenase RLL* (*LOX-RLL*)	Rice (*Oryza sativa* ssp. *japonica* cv Nipponbare) */* * M. oryzae*	positive	[140]
164 differentially expressed lncRNAs were identified in response to infection */* genes related to kinase activity, phytohormone regulation, and cell wall reinforcement	*Pinus radiate* */* *Fusarium circinatum*	positive	[141]
lncRNA11254 , a natural antisense transcript (NAT) lncRNA to the *RPP8* gene	*Hevea brasiliensis* */* *Colletotrichum gloeosporioides*	positive	[142]
lncRNA11041 and lncRNA11205 interacting with disease responsive miRNAs	*Hevea brasiliensis* */* *Colletotrichum gloeosporioides*	positive	[142]
a total of 14,525 differentially expressed LncRNAs were identified, including 10,645 upregulated – the target genes of upregulated lncRNAs were enriched in immune-related processes, such as activation of innate immune response, defense response to bacterium, incompatible interaction and immune system process, plant hormone signal transduction, phenylpropanoid pathways.	Walnut (*Juglans regia*) /*Colletotrichum gloeosporioides*	positive	[143]
lncRNA109897 */* serine */* threonine-protein kinase-like protein JrCCR4	Walnut */* *Colletotrichum gloeosporioides*	positive	[144]
lncRNA: XLOC_302848, XLOC_321638, XLOC_113815, XLOC_123624	*Triticum aestivum* */* *Fusarium graminearum*	positive	[145]
two lncRNAs: LNC_006805 and LNC_012667 */* genes involved in phenylpropanoid biosynthesis, phenylalanine metabolism, and ubiquinone and other terpenoid-quinone biosynthesis.	Cucumber */* *Podosphaera xanthii*	positive	[146]
lncRNA4504 */* transcripts of genes related to jasmonic acid (JA) biosynthesis (*SlLOXD*, *SlAOS* and *SlAOC*) and its signal transduction (*SlMYC2* and *SlCOI1*)	Tomato */* *B. cinerea*	positive	[147]

### “Cross-kingdom RNAi”

Another important aspect is the fact that most sRNAs work endogenously, but some can travel across organismal boundaries between hosts and pathogens and silence genes in trans in interacting organisms, a mechanism called ‘’cross-kingdom RNAi’’. During the arms race between fungi and plants, some fungi send sRNA as an effector molecule to plant cells to silence plant resistance genes, while plants also transport sRNA mainly using extracellular vesicles to pathogens to suppress virulence-related genes. Cai et al. reported that *A. thaliana* host cells secreted exosome-like extracellular vesicles to deliver sRNA to the fungal pathogen *B. cinerea*. These sRNA-containing vesicles accumulate at sites of infection and are taken up by fungal cells. Transferred host sRNAs induced silencing of fungal genes critical for pathogenicity [148]. Zhang et al. noted that in response to *V. dahliae* infection (a vascular fungal pathogen responsible for wilt diseases in many crops), cotton plants increased the production of microRNAs 166 (miR166) and miR159 and exported both microRNAs to fungal hyphae for specific silencing of virulence genes (*Ca*^*2+*^*-dependent cysteine protease calpain clp-1* (*Clp-1*) and *isotrichodermin C-15 hydroxylase* (*HiC-15*) genes). *Clp-1* and *HiC-15* transcript levels were indeed downregulated in recycled cotton shreds infected with *V. dahliae*. *Clp-1* and *HiC-15* transcript levels were downregulated in *V. dahliae* hyphae recovered from infected cotton [149]. Another example of a plant microRNA affecting a fungal pathogen is tomato miR1001. The study by Meng et al. demonstrated the regulatory role of this miRNA in the growth and development of *B. cinerea*. The results showed that miR1001 inhibited the virulence of *B. cinerea* in infected plants. In addition, miR1001 had a significant inhibitory effect on the germination of *B. cinerea* conidiospores. The results indicated that miR1001 can directly target the genes *Bcin03g02170.1* and *Bcin10g01400.1*, which encode ATP-dependent metallopeptidase and cysteine-type endopeptidase, respectively, in *B. cinerea* [150].

Aggressive fungal pathogens such as *B. cinerea* and *Verticillium* spp. cause serious crop losses worldwide. *B. cinerea* has been found to deliver small RNA (Bc-sRNA) into plant cells to silence host resistance genes. Such sRNA effectors are mostly produced by *B. cinerea* Dicer-like protein 1 (Bc-DCL1) and Bc-DCL2. Wang et al. found that sRNA expression targeting Bc-DCL1 and Bc-DCL2 in *A. thaliana* and tomatoes silenced *Bc-DCL* genes and impaired fungal pathogenicity and growth, exemplifying bidirectional cross-kingdom RNAi and sRNA trafficking between plants and fungi [151].

Increasing evidence has shown that the regulatory function of ncRNAs plays a role in the association of plants with nonpathogenic symbiotic microorganisms. In research on the beneficial fungal endophyte *Fusarium solani* strain K (FsK), transformed FsK with a hairpin RNA (hpRNA) construct designed to target a reporter gene in its host *Nicotiana benthamiana*, after inoculation with the host plant, showed systemic RNA silencing and DNA methylation of the host reporter gene. The hpRNA was processed by FsK RNAi machinery presumably into 21-24-nt small RNAs that triggered RNA silencing but not DNA methylation in the fungal hyphae. These data suggest that RNAi signals can be translocated by endophytes to hosts and can modulate gene expression during mutualism [152].

### Symbiotic fungi and epigenetic modification in plants

In addition to pathogenic fungi, a large proportion of fungal endophytes do not negatively affect the host plant (commensalism) or have a positive effect on plants (mutualism). These types of interactions are called symbiosis. Furthermore, the symbiotic relationship between plant roots and fungi is known as mycorrhiza. Symbiotic endophytes can counteract the development of host plant pathogens, for example by inducing host defense mechanisms or producing compounds that inhibit the growth of other microorganisms. To create and maintain symbiosis, constant communication between the mycobiome and the host plant is required. Furthermore, the plant will have to distinguish whether the microorganism is a friend or a foe. Environmental signals can induce epigenetic regulations that can modulate the host plant’s interaction with microorganisms or trigger defense [153–156].

Endosymbiotic relationships can be controlled by DNA methylation. Varga and Saulsbury carried out an experiment using *Geranium robertianum* in symbiosis with the arbuscular mycorrhizal fungus *Funneliformis mosseae*. This study demonstrated that colonization by an arbuscular mycorrhizal fungus can influence DNA methylation levels in hosts [157]. Moreover, the appropriate methylation status of the host’s DNA is important to create and regulate symbiotic interactions. Vigneaud et al. showed that host DNA hypomethylation limits the formation of ectomycorrhizas on the example of *Populus* spp. and the fungus *Laccaria bicolor* [158]. Research by Hubbard et al. showed that in the endosymbiotic seed-fungus relationship, colonization of the endophytic fungus SMCD 2206 was associated with changes in DNA methylation in wheat subjected to drought stress. Such epigenetic changes can be associated with increased plant resistance to drought. DNA methylation patterns observed in drought-stressed wheat seedlings co-cultured with SMCD 2206 resembled those of the unstressed control [159]. Research by Forte et al. on the mutualistic interaction between Epichloë sp. LpTG-3 strain AR37 and *L. perenne* showed that the presence of the endophyte led to a decrease in DNA methylation across genomic features in the host, with differentially methylated regions primarily located in intergenic regions and CHH contexts [160]. Etemadi et al. investigated the role of miR393, targeting several auxin receptors, during the colonization of *Solanum lycopersicum*, *M. truncatula* and *O. sativa* roots by arbuscular mycorrhizas. The results showed that miR393 is a negative regulator of arbuscule formation by hindering auxin perception in cells containing arbuscules [161]. Endosymbiosis of legumes and arbuscular mycorrhizal fungi is regulated by NSP2, which is a target of microRNA171h (miR171h). The spatiotemporal expression of miR171h and *NSP2* is closely related to the nutritional status of the plant [162]. Results of research on *M. truncatula* conducted by Lauressergues et al. suggest that there is a regulatory mechanism involving miR171h-mediated negative regulation of NSP2, triggered by lipochito-oligosaccharides. This mechanism prevents excessive colonization of roots by arbuscular mycorrhizal fungi [163]. Furthermore, a microRNA targeting *NSP2* (miR171h) is also rapidly induced by cytokinins and then shows an expression pattern anticorrelated with *NSP2* [164]. Another *M. truncatula* miRNA, miR169a, was found to target *MtHAP2* expression to control nodule cell differentiation and nodule development [165]. Analysis of the *Phaseolus vulgaris* genome allowed the identification of a set of small RNAs potentially important for regulating symbiosis. Among them, six, including miR-RH82, were differentially expressed in response to treatment with nodulation agents and may play a critical role during the early stages of symbiosis [166]. Studies in maize have shown that lncRNAs are involved in beneficial interactions between plants and the microfungus *Rhizophagus irregularis*. 63 lncRNAs were differentially expressed. The target genes of differentially expressed lncRNAs were mainly related to transmembrane transport of phosphate ions, the cellular response to potassium ion deficiency, and lipid catabolic processes [167]. It appears that suppression by epigenetic modifications of the plant defense system during symbiosis, as well as epigenetic changes in the symbiont genome, is crucial for promoting symbiosis and maintaining beneficial interactions. However, this still needs to be explored through further research.

### Epigenetic memory

The immune memory is associated with the process of preparing cells (priming), consisting in strengthening the defensive response of plants previously in contact with the stress factor, activated only in response stress. Priming works at the phenotypic level do not change the DNA sequence, and can be reversible. The plant, under the influence of stress, corresponds to changes in gene expression: faster/earlier, more strongly, or expression of genes not expressed in a healthy plant. Epigenetic changes provide plants with immune memory [19, 44, 168, 169]. Among forms of epigenetic immune memory of plants, we can specify somatic memory (within the life cycle of a plant), intergenerational memory (stable for the first generation), and transgenerational memory (stable for at least two generations) [170].

**Fig. 6:**
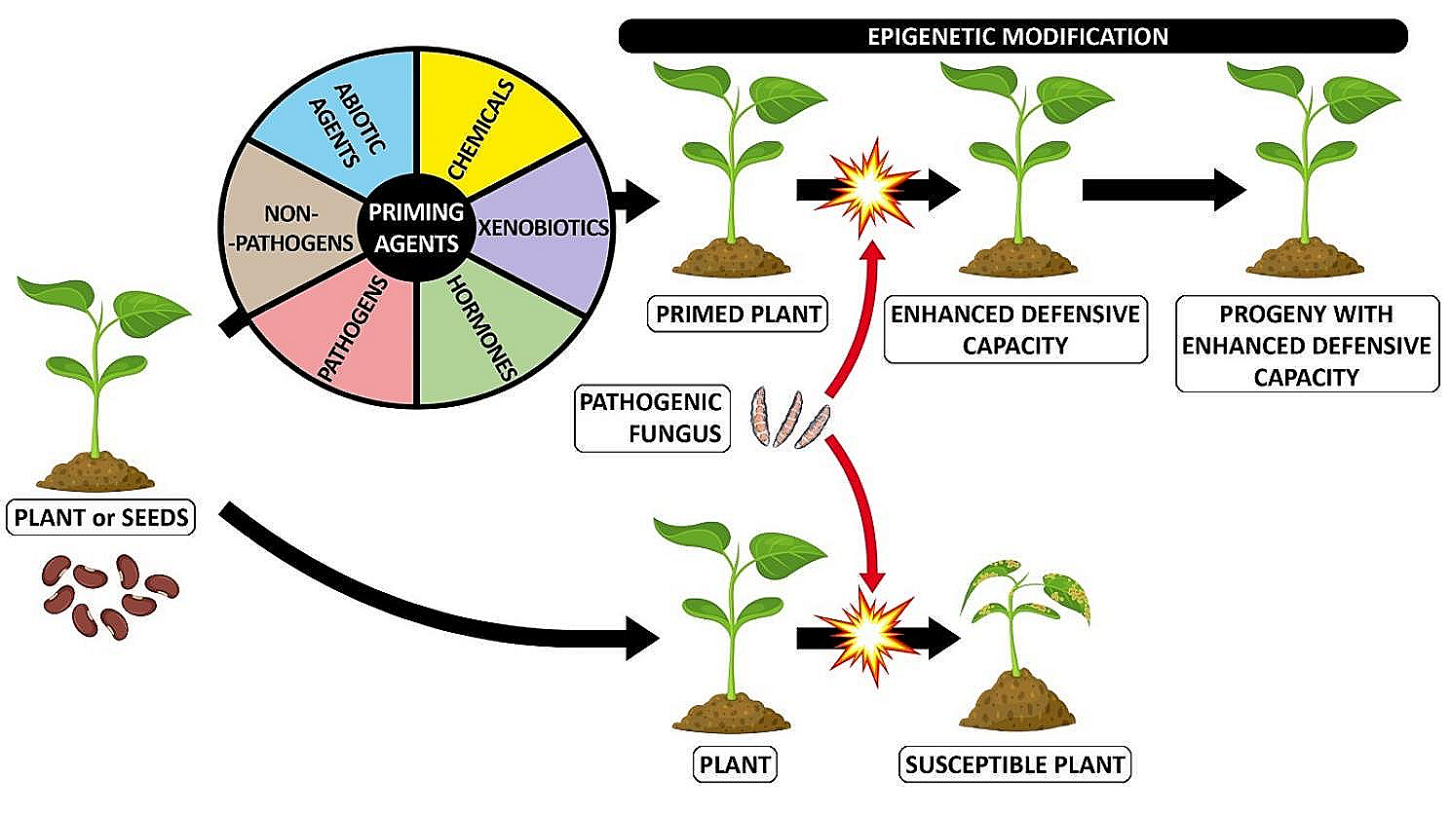
Priming of plant defense in response to fungal attack. The process of priming seeds and plants using various priming agents, including chemical compounds, xenobiotic chemicals, hormones, microbes (both nonpathogenic and pathogenic strains), and abiotic stress, induces epigenetic modifications. These modifications play a significant role in augmenting the defense mechanisms of primed plants when exposed to pathogenic fungi. Moreover, these enhanced defense capabilities can potentially be inherited by subsequent generations of primed plants

Epigenetic memory leading to increased resistance to fungal pathogens can be induced by priming agents, including chemical compounds: β-aminobutyric acid (BABA), xenobiotic chemicals: benzothiadiazole (BTH) or (R)-β-homoserine (RBH), hormones (SA, JA, MeJA), microbes: *Bacillus*, *Pseudomonas*, plant-growth-promoting rhizobacteria (PGPR): *Trichoderma* spp., nonpathogenic strains of *Fusarium* spp., *Piriformospora indica*, and arbuscular mycorrhizal fungi (AMF) from the genus *Glomeromycota* and abiotic stress (salt, cold, heat) [7, 171, 172]. Seeds and plants could be primed. Seed priming increases seedlings’ tolerance of environmental stresses. Primed plants respond faster and/or more strongly to recurring defense stimuli. Some priming states are relatively short-term and disappear within a few days, while others are long lasting and can even be transmitted between plant generations (Fig. 6).

Exposure of plants to sub-lethal levels of salt, cold, or heat can enhance their resistance to infection by pathogens. In such cross-priming, PTI-responsive genes are enriched by histone acetylation associated with their transcriptional activation [23].

The existing literature provides a wealth of information on plant defense priming and immune memory, primarily focusing on the resistance of plants to bacterial or oomycete pathogens. However, there is a noticeable lack of information regarding the specific epigenetic mechanisms involved in these processes when it comes to fungal pathogens. In this review our purpose was to exclusively present examples demonstrating that seed or plant priming operates through epigenetic mechanisms, thereby enhancing plant resistance to fungal pathogen infections.

Epigenetic mechanisms underlying priming are not well understood. Catoni et al. investigated epigenetic changes in tomato plants primed for pathogen resistance by treatment with β-aminobutyric acid. Genomes of plants treated with BABA showed a significant reduction in global cytosine methylation, especially in CHH sequence contexts. Differentially methylated region (DMR) analysis showed that the CHHs in the DMR were almost exclusively hypomethylated. A large number of DMRs have been reported in gene promoters that are differentially expressed in response to infection with *B. cinerea*. However, most genes that showed priming did not contain DMR, and the overall distribution of methylated cytosines in priming genes was not altered by BABA treatment. It is possible that the BABA treatment of tomato seedlings causes characteristic changes in genome-wide DNA methylation, and that CHH hypomethylation only affects a minority of genes showing primed responses to pathogen infection. Probably methylation may affect priming via in-trans regulation, acting at a distance from defense genes, and by targeting a smaller group of regulatory genes controlling stress responses [173].

A lot of research has focused on seed priming [174], but the process can be successfully performed on whole plants. A good example is the study of Wojtasik et al., where flax seedlings were treated with the non-pathogenic strain *F oxysporum* Fo47 to increase resistance to the pathogenic strain of *F oxysporum* L. f. sp. *lini*. Two contrasting effects on the levels of methylation in flax were detected for both types of *Fusarium* strain infection: genome-wide hypermethylation and hypomethylation of resistance-related genes (*β-1,3-glucanase* and *chitinase*). Despite the differences in methylation profile, the expression of these genes increased. The peak of demethylation correlated with the alteration in gene expression induced by the non-pathogenic strain. In the case of pathogen infection, the expression peak lagged behind the gene demethylation. Plants pretreated with the non-pathogenic strain memorized the hypomethylation pattern and then reacted more efficiently upon pathogen infection [175].

Not only DNA methylation can affect priming but also processes related to non-coding RNAs play an important role. Wang et al. studied the small RNAs and transcriptome changes in maize leaves that were systemically induced by seed treatment with *Trichoderma harzianum* (strain T28) against *Cochliobolus heterostrophus* infection in leaves. Analysis of the sequencing data showed that genes involved in the plant hormone signal transduction pathway and the oxidation-reduction process were significantly enriched. In addition, 15 pairs of miRNA-mRNA interactions that played a role in *T. harzianum*-induced maize resistance to *C. heterostrophus* were identified; miR390, miR169j, miR408b, miR395a/p and the novel miRNA miRn5231 were most involved in the induction of maize resistance [176].

## Conclusion

In this review, we have presented examples of how plants use epigenetic mechanisms to fight fungi, demonstrating how powerful and necessary this weapon is. All these mechanisms – DNA methylation, covalent histone modifications, ATP-dependent chromatin remodeling and ncRNAs – can operate independently or be coupled. Epigenetic mechanisms are activated in plants not only during pathogenic infection but also during the symbiotic relationship of plants with endophytic fungi, which aims to maintain beneficial interactions and promote symbiosis. Furthermore, epigenetic mechanisms are integral to both plant priming and the formation of stress-induced environmental epigenetic memory.

## Data Availability

All data generated or analyzed during this study are included in this published article.

## Abbreviations

A: Adenine
AGO: Argonaute protein
AMF: Arbuscular mycorrhizal fungi
AtELP: Elongator Subunit
BABA: β-aminobutyric acid
Bgt: *Blumeria graminis* f. sp. *tritici*
BIT: 1,2-benzisothiazol-3(2 h)-one,1, 1-dioxide
BRM: Brahma protein
BTH: Benzothiadiazole
CC: Coiled-coil
C: Cytosine
CHD: Chromodomain helicase DNA-binding
CHR: Chromatin remodeler
COI1: Coronatine insensitive 1
CRC: Chromosome remodeling complex
CWDE: Cell wall degrading enzyme
DAMP: Damage-associated molecular pattern
DDM1: Decreased DNA Methylation 1
DME: DEMETER (5-methylcytosine DNA glycosylase/demethylase)
DML2: DEMETER-Like protein 2
DMR: Differentially methylated region
dsRNAs: Double-stranded RNAs
EIN2: Ethylene-insensitive 2 gene
ER: ERECTA kinase
ET: Ethylene
ETI: Effector-triggered immunity
G: Guanine
HAT: Histone acetyltransferase
hc*-*siRNAs: Heterochromatic small interfering RNAs
HDAC: Histone deacetylase
HR: Hypersensitive response
INO80: Inositol auxotrophy 80
ISR: Induced systemic resistance
ISWI: Imitation switch
JA: Jasmonic acid
lncNAT: Long noncoding natural antisense transcript
lncRNAs: Long non-coding RNAs
lncTARs: Long noncoding transcriptionally active regions
LTR: Long terminal repeat
MAMP: Microbial associated molecular pattern
MAPK: Mitogen-activated protein kinases
MET: Methyltransferase
miRNA: MicroRNA
MITE: Tourist-miniature inverted-repeat transposable element
Mla: Mildew resistance locus
MOS1: Modifiers of snc1
NB-LRR: Nucleotide-binding leucine-rich repeat
NB: Nucleotide-binding
NF: Nuclear factor
NLR: Leucine-rich domain protein
NLRs: NB-LRR-containing R proteins
NPR1: Non-expressor of PR genes
nt: Nucleotide
OGs: Oligogalacturonides
PAMP: Pathogen-associated molecular pattern
PG: Polygalacturonase
phasiRNA: Phased small interfering RNA
PL: Pectin lyase
PME: Pectin methylesterase
Pol: Polymerase
PR genes: Pathogenesis-related genes
PRR: Pattern recognition receptor
PTI: Pattern-triggered immunity
rar1: For Mla12 resistance1
RBH: (R)-β-homoserine
RdDM: RNA-directed DNA methylation
RLKs: Receptor-like kinases
rom1: Restoration of Mla resistance1
ROS: Reactive oxygen species
RT-PCR: Reverse-transcription polymerase chain reaction
SA: Salicylic acid
SAR: Systemic acquired resistance
siRNAs: Small interfering RNAs
SNC1: Suppressor of npr1-1, constitutive1; one of the R genes
SOD: Superoxide dismutase
sRNAs: Small RNAs
SWI/SNF: Switch/Sucrose on-fermentable
SYD: Splayed protein
TE: Transposable element
TF: Transcription factor
TIR: Toll/Interleukin1 receptor-like
T: Thymine
YDD: YODA DOWNSTREAM
